# New Guidelines for the Management of Chest Pain: Lessons From a Recent Audit in Tauranga, New Zealand

**DOI:** 10.4021/cr116w

**Published:** 2012-01-20

**Authors:** Andrew R Chapman, Stephen J Leslie, Derek K Sage

**Affiliations:** a Highland Heartbeat Centre, Raigmore Hospital, Inverness, IV2 3UJ, UK; b Tauranga Emergency Department, Cameron Road, Tauranga 3110, New Zealand

**Keywords:** Chest pain, Oxygen therapy, Medical audit

## Abstract

**Background:**

Protocol based care is known to improve the outcomes of patients admitted with recent onset chest pain. The aim of this clinical review was to investigate chest pain management, using newly published guidance from NICE, in the emergency department of a regional hospital in New Zealand.

**Methods:**

All admissions with chest pain during the period of September-October 2010 were identified retrospectively (n = 599), and a sufficiently powered random sample (n = 120) taken. Relevant data was identified from patient notes, including basic demographics and specific management details.

**Results:**

One hundred and eighteen patients were analysed (M = 65, F = 53), 99.2% received an ECG on admission, yet only 59.3% of patients had documented evidence of a repeat ECG, with admissions during the day less likely to receive one compared to those admitted overnight (51.5% vs 69.2%, P = 0.04). Younger patients (< 39 years) appeared less likely to receive aspirin than older patients (38.9% vs 80.0%, P = 0.06), 21.3% of patients failed to receive 300 mg aspirin, and 45.6% of patients received oxygen despite normal saturations.

**Conclusions:**

Despite good performance in a number of areas, this clinical review has highlighted that some standards, such as repeat ECGs, administration of aspirin therapy and appropriate use of oxygen are not being met in all patients with chest pain. A chest pain management pathway is to be implemented for all relevant admissions, to ensure that essential aspects of care are not missed. Timely dissemination and implementation of new clinical guidelines remains a challenge in clinical practice.

## Introduction

Chest pain is a common and often non-specific symptom that can be caused by a number of underlying conditions, one of the most serious being acute myocardial infarction. Whilst only around a third of these presentations are cardiac in origin [1], the potential severity of myocardial ischaemia necessitates rapid assessment and management.

In New Zealand, cardiovascular disease is the leading cause of mortality, with coronary artery disease responsible for 23% of all deaths in 1999. Over 52% of these deaths were due to myocardial infarction [2]. Whilst rates of coronary artery disease are thought to be in decline, research has shown that Maori individuals are more likely to die from a myocardial infarction than age stratified European counterparts, with men and women at a 1.6 and 4.2 times increased risk, respectively [3, 4].

Furthermore, mortality associated with coronary artery disease in Maori’s under the age of 65 is almost three times that found in Europeans of a similar age [4]. Cardiovascular disease is therefore a major public health concern in New Zealand, and campaigns to reduce the impact of modifiable risk factors such as smoking are widespread.

The National Institute for Health and Clinical Excellence (NICE) is an independent UK organisation which provides national guidance on promoting health and preventing and treating poor health. They published updated guidelines for the management of recent onset chest pain in March 2010 [5]. This document is intended to streamline the management of new presentations with chest pain, and ensure that clinicians are up to date with current literature. The key points of this new guideline are summarised in Table 1.

**Table 1:** Adapted From NICE Guideline 95 [5]

Summary of NICE Guidance regarding management of acute chest pain presentations
Take a resting 12-lead ECG as soon as possible. When a patient is referred, send the results prior to arrival provided this does not delay transfer.
Do not exclude an acute coronary syndrome (ACS) when patients have a normal resting 12-lead ECG.
Do not routinely administer oxygen. Monitor oxygen saturation using pulse oximetry as soon as possible, ideally pre-admission.
Only offer supplementary oxygen to:
-People with SpO_2_ <94% who are not at risk of hypercapnic respiratory failure, with a target SpO_2_ of 94-98%
-People with chronic obstructive pulmonary disease who are at risk of hypercapnic respiratory failure, with a target SpO_2_ of 88-92%, until blood gas available.

The College of Emergency Medicine (UK) established standards for the treatment of suspected acute coronary syndrome [6]. In their most recent publication, they present time-frames for assessment or intervention which emergency departments should strive to achieve. They suggest that 90% of all patients with chest pain should have aspirin and an ECG within 10 minutes of arrival at an emergency department, 75% of patients in pain should receive analgesia within 30 minutes, and 90% within an hour.

In recent years, cost reduction strategies have led to emergency departments worldwide admitting only those patients deemed essential for monitoring, and reducing a patient’s length of stay in hospital where possible, whilst maintaining a high standard of care. The focus of emergency medicine has now shifted to the development and implementation of high quality protocol based care, to ensure that all patients are screened in a systematic manner, and management is optimal.

Despite this, it is estimated that between 2 - 5% of acute myocardial infarction patients are discharged inappropriately [7]. These individuals often have a very poor outcome, and in addition, the discharge of patients later found to have suffered a heart attack is a leading cause of malpractice lawsuits each year [8]. It is essential to ensure that the highest standards of care are maintained, as such, audit is an important asset which may be used to implement quality evidence based medicine.

Whilst there are New Zealand based guidelines for the management of established acute coronary syndrome, there was no available comparable guideline for the initial management of recent onset chest pain. The aims of this clinical review were to assess baseline clinical standards in a regional emergency department in New Zealand, and to ascertain whether current practice had adapted to incorporate recent published developments in chest pain management.

## Methods

### Setting

Tauranga Hospital has one of the largest emergency departments in New Zealand, treating 45000 patients each year, including around 4000 presentations with chest pain.

### Patient selection

All admissions to the emergency department of Tauranga Hospital between 01/09/10 and 31/10/10 were screened for the presenting complaint of ‘chest pain’ using the medical records database. Patients were included if they had either ongoing chest pain, or recent chest pain (< 12 hours) which was now resolved. Those under the age of 18 were excluded, as were presentations which were known to be non-cardiac at an early stage and therefore not managed on an appropriate pathway for audit.

### Sample size

This search generated records of 599 patients that fitted the inclusion criteria. After the population was identified, a sample size calculation was performed. As this audit is based on standards of 100%, the likelihood of a positive response for measured criteria is high, therefore a response distribution of 90% was chosen. To detect a statistical effect with a confidence level of 95%, and 5% margin of error, the minimum sample size required is 113. Using the statistical package for social sciences (SPSS) version 18.1, a simple randomisation scheme was employed, and 120 patients were identified.

### Data handling and statistical analysis

Data was obtained from patient notes by the principal researcher and collected using an audit proforma consisting of 32 different variables (Appendix 1). Results were then compiled in a spread sheet. Data was handled using SPSS, and assessed for normality. Appropriate statistical tests were employed as detailed in the results section. The level of statistical significance was deemed (P < 0.05) for all calculations.

### Confidentiality

All patients were allocated a number, and data was recorded anonymously to ensure confidentiality was maintained. This research was exempt from formal ethical committee procedure, however, it was approved by the Research Manager of the Bay of Plenty District Health Board.

## Results

From the original population of 599, 120 patients were identified at random. Two cases identified by the randomisation scheme were not eligible for inclusion as their presentations were not considered to be cardiac in origin at time of admission, leaving a total number of 118 eligible cases (53 females and 65 males).

Ages of those in the sample were found to be normally distributed. Women were older than men (64.9 ± 18.5 vs 61.6 ± 17.2, P = 0.24). Time of admission was also found to be normally distributed within the sample. Results highlighting the performance of the department compared to NICE standards are shown in Table 2.

**Table 2:** Results Comparing Tauranga Hospital to NICE Standard

	Tauranga Hospital % concordance and (number/all eligible)
Initial Assessment	
Current Pain Status	100%
Time of Onset	100%
Resting ECG	99.2% (117/118)
Pain relief (if required)	91.3% (74/81)
Aspirin (if not allergic)	78.7% (74/94)
Appropriate O_2_ usage	54.4% (31/57)
Cardiac Marker tested	97.5% (115/118)
Monitoring	
Pulse oximetry	98.3% (116/118)
Pulse	99.2% (117/118)
Blood Pressure	100%
Pain	94% (111/118)
Repeat ECG	59.3% (70/118)
Telemetry	57.6% (68/118)
Further Assessment	
Pain Characteristics	100%
Associated symptoms	93.2% (110/118)
Cardiovascular History	98.3% (116/118)
Risk Factors	77.1% (91/118)
Previous Episodes	100%
Full Examination	100%

All aspects investigated correlate to NICE guideline 95, and established concordance targets are 100%.

**Figure 1:**
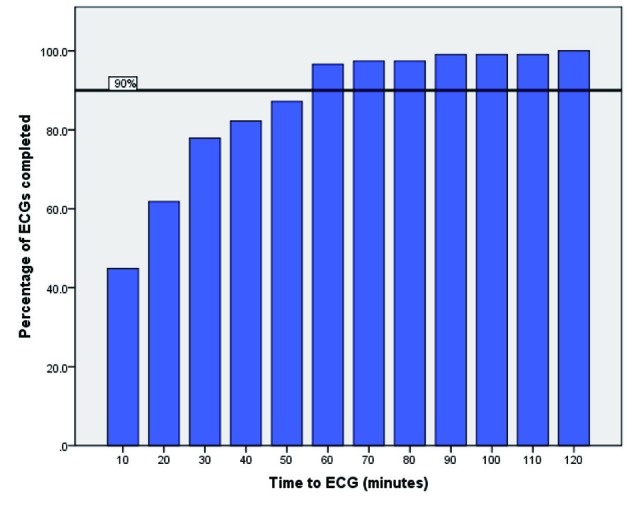
This bar graph highlights the time to initial ECG within the emergency department, versus the total number of completed ECGs. The College of Emergency medicine target of 90% ECG completion within 10 minutes is shown in black.

**Figure 2:**
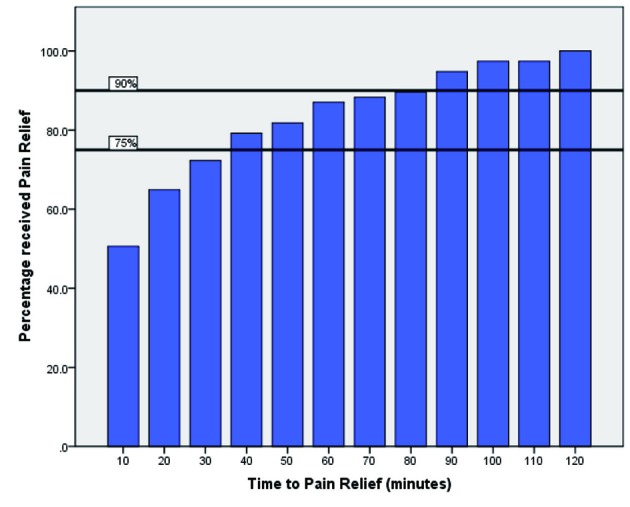
This bar graph depicts time to administration of pain relief after admission, versus the total percentage of patients requiring pain relief. The College of Emergency medicine targets of 75% pain relief within 30 minutes, and 90% within an hour, are shown in black.

**Figure 3:**
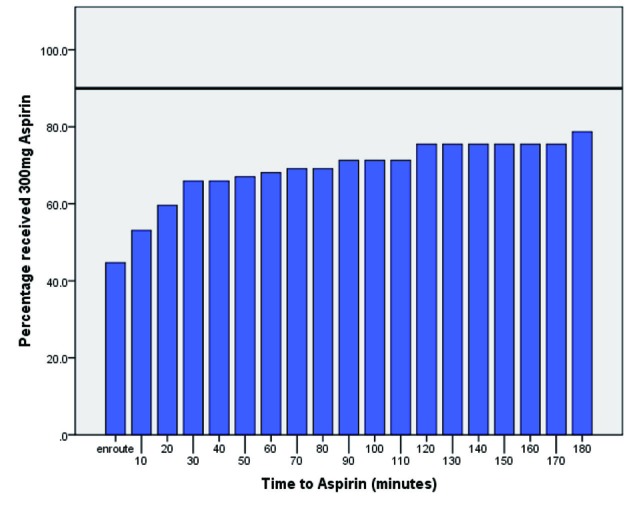
This bar graph depicts time to aspirin prescription from admission. The College of Emergency Medicine target of 90% prescription to those eligible is highlighted in black.

Results comparing the department to the College of Emergency Medicine (UK) guidelines for pain management, aspirin prescription and time to ECG are summarised in Figures 1, 2 and 3.

Time of admission was investigated as a potential influencing factor on the management of patients with chest pain. As hospitals have fewer staff at night, it was hypothesised that those admitted outside of normal working hours (17:30 - 08:00) would wait longer for clinical management and investigation. Further investigations sought to establish the effect of patient age on management decisions. Formal statistical testing was applied, with results as shown in Table 3.

**Table 3:** Highlights Investigation of Time of Admission as a Potential Influence on the Management of Patients With Chest Pain

		P value
Time of Admission	Time to ECG †	0.784
	Time to Pain Relief †	0.342
	Time to Venepuncture †	0.783
	Pain Monitoring ‡	0.352
	Telemetry ‡	0.177
	Repeat ECG ‡	0.039**
		
Patient Age	Aspirin use	0.067*

† investigated with Mann Whitney U-Test; ‡ investigated with Fisher’s exact test for contingency tables; *denotes statistical significance at the 10% confidence level;**denotes statistical significance at the 5% confidence level.

## Discussion

This clinical review has identified several areas for discussion. Compliance with recent NICE standards was noted in establishing pain status and pain characteristics, eliciting previous episodes of chest pain, recording blood pressure, and completing a detailed physical examination. Furthermore, 99.2% of patients received an ECG during their presentation to the emergency department. It is likely that these measures would identify the vast majority of patients presenting with an acute coronary syndrome.

However, some areas for improvement were identified. Risk factor documentation was incomplete in a proportion of patients and a repeat ECG was frequently not undertaken. Aspirin was generally under prescribed and oxygen therapy was given outwith the recent guidelines. Trends involving time of admission, patient age and management decisions were also identified, and may require further exploration.

### ECG and telemetry

An ECG was recorded in the vast majority of patients in this cohort (99.2%). However, a single normal 12 lead resting ECG does not exclude a diagnosis of ACS, and NICE guidance recommends that all patients should have serial ECGs and continuous monitoring with telemetry when clinically appropriate. This study has found that only 59.3% of patients admitted with chest pain received more than one ECG, and only 57.6% were continuously monitored with telemetry. Serial ECGs improve the outcomes of patients later diagnosed with both ACS and MI, and their use is recommended in high risk patients [9].

It has been suggested that failure to repeat ECGs may lead to an increase in the number of missed cardiac diagnoses, however, there was no evidence this happened during the study. It is possible that the actual number of repeat ECGs was higher than found in this study, yet due to a combination of poor recording in the notes, and ECGs not being filed, the numbers appear lower.

The local policy on continuous telemetry suggests that those diagnosed with either AMI or unstable angina should be monitored, excluding those without a definitive diagnosis. On two occasions, telemetry was indicated but clinicians commented that it was unavailable. Unfortunately these comments are likely to be a true reflection of the financial constraints on the emergency department.

### Initial therapy

Aspirin is indicated in the initial management of patients with suspected cardiac chest pain. However in this cohort the use of aspirin appeared to be poor. Aspirin is frequently given en route by paramedic staff, and it is possible that this was not always documented. Furthermore, after discussion with staff members, it appears that some clinicians favour the prescription of clopidogrel over aspirin in the acute setting.

No systematic reviews or randomised controlled trials studying the use of aspirin versus clopidogrel in patients with acute current chest pain could be identified in the literature. NICE base their guidance on a cohort study assessing the efficacy of aspirin when given pre or post hospital admission. A lower mortality rate was found in patients that received aspirin pre admission compared to post admission at 7 days, (2.4% versus 7.3%, P *=* 0.002) [10], and this remained significant at 30 day follow up. Pre hospital aspirin reduced the risks of asystole (P < 0.001), resuscitation (P < 0.001) and ventilation (P < 0.002).

NICE recommend that other anti-platelet agents, such as clopidogrel, should only be given after patients have had an initial assessment which has refined the likely diagnosis, and they suggest that those diagnosed with AMI or ACS should be managed in line with other relevant guidelines [5].

The Scottish Intercollegiate Guidelines Network (SIGN) guidelines state that patients with acute ECG changes should receive a combination of both aspirin and clopidogrel, as this improves patient outcomes despite an increase in secondary bleeding [11].

Oxygen is indicated in patients with low oxygen saturations (< 94%) on air. Recent guidelines from NICE and the British Thoracic Society agree that supplementary oxygen has not been shown to be beneficial to these patients, and may indeed be harmful [5, 12]. In those with no risk of hypercapnic respiratory failure, the target saturation should be 94 - 98%, and in those who are at risk, a target of 88 - 92% is appropriate, until an arterial blood gas is available.

However, clinical practice has been slow to change in this regard and routine use of oxygen therapy persists despite these guidelines. The rate of oxygen prescription in those with normal saturations in this cohort was high, with 45.6% of individuals receiving oxygen inappropriately. Education of all members of staff may help to address this issue, improve patient care and allow resources to be allocated elsewhere.

### Time of admission

Published research has shown that patients with myocardial infarction admitted to hospital overnight or at the weekend are managed less efficiently than daytime admissions [7, 13, 14]. This is of particular interest when time targets are established. This association may be a direct effect of staffing differences between day and night shifts.

In this current study the only significant correlation between time of admission and management was the use of the repeat ECG. Results show that patients admitted during the day were less likely to receive a repeat ECG than those admitted overnight (P = 0.032). It is possible that due to a larger number of general attendees to the emergency department during normal working hours, there is an increased demand for routine ECGs, and therefore admissions with chest pain were not monitored as often as they should be. However, it is also possible that repeat ECGs were performed, but were not recorded or filed within the medical record. Regardless, this would seem to imply that perhaps additional staffing is required during busy periods.

Further published research has highlighted associations between patient age and management of chest pain. Younger patients admitted with chest pain are significantly less likely to receive therapeutic aspirin (300 mg), than older counterparts [15].

Whilst we have also found trends between patient age and aspirin prescription, these narrowly failed to reach significance. It is likely that this result is due to the small numbers available for statistical testing once stratified into age appropriate groups, and an adequately powered study is likely to have confirmed such a relationship. There is limited evidence to suggest that this lack of prescription is detrimental to patient care, and indeed this may be clinically appropriate decision making. As outcome data was not available within our study, this is unknown, however it would be interesting to explore this relationship in future work.

### Limitations

This was a retrospective audit, however the use of an audit data collection tool ensured that a complete dataset was obtained, and maintained consistency in recording data between patients. There was a low rate of exclusion within the study cohort, as only two of the original 120 identified were omitted from analysis. This improves the validity of the results.

As this was single centre study the results may not be fully generalisable, however, the setting in a typical busy general hospital is likely to be representative of other centres.

### Implications for future practice

In order to standardise the assessment and treatment of patients with acute chest pain, a new admission proforma has been implemented into the department. The current general admission document used within the emergency department does not contain a pre-written checklist section to be completed regarding risk factors, and this has also been updated.

As yet, there have been no published audit findings which discuss the uptake of new guidance on the management of recent onset chest pain, in particular the delivery of oxygen, and this paper highlights issues that still need to be addressed. As with all changes in medical practice, these guidelines will take time to become fully implemented. This early clinical review serves to raise awareness of the challenges which must be overcome in order to comply with new standards.

### Conclusions

Despite good performance in a number of areas, this clinical review has highlighted that some standards are not being met in patients with chest pain. Clinicians must be aware of up to date guidance regarding oxygen therapy in chest pain, and aspirin should be used in all eligible patients when clinically appropriate. Serial ECGs must be used as a single 12 lead ECG does not exclude a diagnosis of acute coronary syndrome.

## References

[1] Capewell S, McMurray J (2000). “Chest pain-please admit”: is there an alternative?. A rapid cardiological assessment service may prevent unnecessary admissions. BMJ.

[2] World Health Organisation (WHO) http://www.who.int/whosis/mort/profiles/mort_wpro_nzl_newzealand.pdf.

[3] Tipene-Leach D, Stewart A, Beaglehole R (1991). Coronary heart disease mortality in Auckland Maori and Europeans. N Z Med J.

[4] Bramley D, Riddell T, Crengle S, Curtis E, Harwood M, Nehua D, Reid P (2004). A call to action on Maori cardiovascular health. N Z Med J.

[5] NICE (2010). Chest pain of recent onset: Assessment and diagnosis of recent onset chest pain or discomfort of suspected cardiac origin. Clinical Guideline.

[6] College of Emergency Medicine http://www.collemergencymed.ac.uk/.

[7] Collinson PO, Premachandram S, Hashemi K (2000). Prospective audit of incidence of prognostically important myocardial damage in patients discharged from emergency department. BMJ.

[8] Schull MJ, Vermeulen MJ, Stukel TA (2006). The risk of missed diagnosis of acute myocardial infarction associated with emergency department volume. Ann Emerg Med.

[9] Fesmire FM (2000). Which chest pain patients potentially benefit from continuous 12-lead ST-segment monitoring with automated serial ECG?. Am J Emerg Med.

[10] Barbash IM, Freimark D, Gottlieb S, Hod H, Hasin Y, Battler A, Crystal E (2002). Outcome of myocardial infarction in patients treated with aspirin is enhanced by pre-hospital administration. Cardiology.

[11] Scottish Intercollegiate Guidelines Network (SIGN) Acute coronary syndromes: A national clinical guideline.

[12] The British Thoracic Society Emergency Oxygen Use in Adult Patients.

[13] Schull MJ, Vermeulen M, Slaughter G, Morrison L, Daly P (2004). Emergency department crowding and thrombolysis delays in acute myocardial infarction. Ann Emerg Med.

[14] Magid DJ, Wang Y, Herrin J, McNamara RL, Bradley EH, Curtis JP, Pollack CV (2005). Relationship between time of day, day of week, timeliness of reperfusion, and in-hospital mortality for patients with acute ST-segment elevation myocardial infarction. JAMA.

[15] Takakuwa KM, Shofer FS, Hollander JE (2010). Aspirin administration in ED patients who presented with undifferentiated chest pain: age, race, and sex effects. Am J Emerg Med.

